# A20 EARLY-LIFE FUNGAL COLONIZATION MEDIATES HOST METABOLISM AND WHITE ADIPOSE TISSUE INFLAMMATION IN MICE

**DOI:** 10.1093/jcag/gwac036.020

**Published:** 2023-03-07

**Authors:** M W Gutierrez, E van Tilburg Bernardes, K Kalbfleisch, F Chleilat, M -C Arrieta

**Affiliations:** 1 Physiology & Pharmacology; 2 Pediatrics, University of Calgary, Calgary, Canada

## Abstract

**Background:**

The gut microbiome has been linked to metabolic diseases including obesity, however the role of fungi (mycobiome) remains understudied. Recently, fungal taxa have been correlated with obesity in humans, though their causal contribution to obesity development, especially in the context of early life, is unknown. Obesity has been associated with metabolic inflammation, including alterations to the white adipose tissue (WAT) immune landscape, which has been shown to be influenced by the microbiome. Given the potent modulation of host immunity by the mycobiome, it is plausible that it also influences WAT inflammation.

**Purpose:**

This research aimed to explore the role of early-life colonization by specific fungal taxa in obesity development and WAT inflammation.

**Method:**

Gnotobiotic mice were colonized from birth with 12 mouse-derived bacteria (Oligo-MM12) alone or in combination with *Candida albicans* or *Rhodotorula mucilaginosa*. Mice were weaned onto a control or high-fat-high-sugar diet (HFHS) and evaluated at 12 weeks for metabolic and associated inflammatory outcomes.

**Result(s):**

*C. albicans* colonization reduced body weight in mice fed control diet, and induced resistance to weight and adiposity gain in mice fed HFHS. In contrast, *R. mucilaginosa* colonization was associated with increased adiposity in mice fed control diet, and elevated glycemia and LDL-cholesterol in mice fed HFHS. Fungal colonization had a broad impact on immune cells in white adipose tissue. *C. albicans* colonization was associated with increased adipose tissue inflammation with elevated Th1, Th17, ɣδT cells, ILC1, NK cells, cDC1 and neutrophils independently of diet. Additionally, vascular associated macrophages (VAM), CX3CR1+ macrophages and DCs, and ILC3 were elevated in mice fed control diet, while mice fed HFHS displayed elevated Th2, CD8+ T cells and eosinophils. In contrast, *R. mucilaginosa* colonized mice displayed decreased adipose tissue B cells and increased VAMs when fed control diet, and increased CX3CR1+ DCs when fed HFHS. Interestingly, *C. albicans* colonization was associated with increased relative expression of *mPgc1⍺* in white adipose tissue of HFHS fed mice, indicative of enhanced mitochondrial biogenesis.

**Conclusion(s):**

Elevated adipose tissue inflammation with *C. albicans* colonization suggests dysfunction of energy storage and may explain the decreased body weight and resistance to diet-induced obesity, while the immune changes in *R. mucilaginosa* colonized mice may exacerbate obesity development. This work revealed that two common fungal colonizers have distinct and striking influences on obesity and metabolic inflammation and prompts for the inclusion of fungi in microbiome studies on host metabolism.

**Please acknowledge all funding agencies by checking the applicable boxes below:**

CIHR, Other

**Please indicate your source of funding:**

Cumming School of Medicine

**Disclosure of Interest:**

None Declared

